# LEDGF/p75 IN interaction inhibitors: *in silico* studies of an old target with novel approach

**DOI:** 10.1186/1471-2334-14-S3-P18

**Published:** 2014-05-27

**Authors:** Amit M Pant, Rupesh V Chikhale, Sunil S Menghani, Pramod B Khedekar

**Affiliations:** 1 Department of Pharmaceutical Sciences, RTM Nagpur University, Nagpur, India

## Background

Despite development in Anti Retroviral Therapy (ART), reports of HIV infection remains in continuous momentum and a cure seems to be imaginary. Raltegravir, an Integrase (IN) inhibitor, provides some life expectancy to patients on salvage therapy. Nowadays, IN inhibitors reported with resistance and shows cross resistance to other drugs in this class. Human Lens Epithelium Derived Growth Factor (LEDGF)/p75 plays a vital role in the HIV life cycle and its importance has been shown in numerous studies. In the LEDGF/p75 IN complex, LEDGF binds to IN at a region other than the catalytic active site. Thus, we tried computationally to approach these IN-LEDGF interaction sites as a novel target in therapy.

## Methods

The computational studies involved protein preparation, ligand preparation and energy minimization, grid generation, docking and analysis of results. A library of 396 molecules were prepared considering a pyrimidine ring as core. These operations were performed using Maestro, Discovery studio and VLife Sciences suites.

## Results

It is known that Ile365 establishes a hydrogen bond with backbone carbonyl group of IN Gln168 whereas Asp366 of LEDGF/p75 forms a hydrogen bond with Glu170, on similar basis it was found that AMP_1071 exhibits hydrogen bonding with Gln95 of one monomer and Gln168, Hie171, and Thr174 of another monomer of IN.

## Conclusion

The designed molecule AMP_1071 shows topological similarity to LEDGF/p75 binding surface. Further antiviral activity, pharmacokinetic and tolerability studies are ongoing. The LEDGF binding inhibitors lacks the cross resistance to any class of ART, possibly making this class as add on to highly active anti-retroviral therapy.

